# Coenzyme Q10 Attenuates Kidney Injury Induced by Titanium Dioxide Nanoparticles and Cadmium Co-exposure in Rats

**DOI:** 10.1007/s12011-024-04469-x

**Published:** 2024-12-21

**Authors:** Yasmina M. Abd-Elhakim, Mohamed M. M. Hashem, Khaled Abo-EL-Sooud, Abeer E. El-metawally, Bayan A. Hassan

**Affiliations:** 1 Department of Forensic Medicine and Toxicology, Faculty of Veterinary Medicine, Zagazig University, Zagazig, 44519 Egypt; 2 Department of Pharmacology, Faculty of Veterinary Medicine, Cairo University, Giza, 12613 Egypt; 3 Pathology Department, Animal Reproduction Research Institute, Giza, 3514805 Egypt; 4 Pharmacology Department, Faculty of Pharmacy, Future University, Cairo, 11835 Egypt

**Keywords:** Kidney, Titanium dioxide nanoparticles, Renal injury, Cadmium, Coenzyme 10, Oxidative stress, Collagen

## Abstract

This study examined the possible defensive role of coenzyme Q10 (CQ10) against the impact of cadmium (Cd) and titanium dioxide nanoparticle (TNP) exposure on rat kidneys. Distilled water (1 mL/rat), corn oil (1 mL/rat), 10 mg CQ10/kg b.wt, 50 mg TNP/kg b.wt, 5 mg Cd/kg b.wt, TNP + Cd, or TNP + Cd + CQ10 was administered orally to seven groups of 70 male Sprague Dawley rats for 60 days. The findings demonstrated that TNP and/or Cd exposure considerably raised serum levels of several renal damage products, disturbed electrolyte balance including sodium, potassium, and calcium, decreased antioxidant enzyme concentration in the kidneys, and elevated malondialdehyde. In addition, rats exposed to TNP and/or Cd had significantly higher levels of renal titanium and Cd. In addition, rats exposed to TNP and/or Cd showed significant histopathological lesions and collagen deposition as revealed by H and E and Masson trichrome staining, respectively. The kidneys were severely damaged by the combined effects of TNP and Cd, although CQ10 greatly mitigated these effects. According to the study, exposure to TNP and Cd can damage the kidneys’ function and structure, especially when combined. However, CQ10 can protect against TNP and Cd’s nephrotoxic effects.

## Introduction

In our day-to-day existence, the kidney’s normal functioning is susceptible to significant harm due to exposure to various environmental contaminants [1]. This exposure gives rise to serious concerns regarding the progression of renal diseases and, consequently, community health. Titanium dioxide nanoparticles (TNP) have been commercialized in large quantities for many products like food, paints, ceramics, and medications in the last several decades [2]. Nevertheless, previous reports showed that TNP induces genotoxicity, cytotoxicity, and inflammation [3]. Specifically, when exposed orally, TNP are mostly absorbed through the intestines and then circulated throughout the body, where they can harm various organs [4]. According to Wang et al. [5], when mice were exposed to TNP, their blood urea and creatinine levels were elevated, and the renal tubule was filled with proteinic liquids. TNP may cause inflammation in the kidneys and a decrease in their function, which in turn causes the accumulation of reactive oxygen species (ROS) [6].

Adding to the straight environmental concerns of NPs like TNP, they commonly co-found with numerous pollutants [7]. Hence, NPs may alter the bioavailability and toxicity of other contaminants, leading to cumulative adverse consequences [8–10]. For example, Deng et al. [11] found that TNP can absorb heavy metal ions. Anthropogenic and naturally occurring processes both contribute to the widespread distribution of cadmium (Cd) in the environment [12]. It is possible to absorb Cd from food, water, and soil. The kidneys are among the many bodily organs that can be damaged by chronic chelation stress [13, 14]. Proteinuria and reabsorption process abnormalities are caused by the accumulation of half of the body load of Cd in the epithelial cells of the proximal tubule [15].

Because of the many possible environmental scenarios involving co-exposure to TNP and Cd, there is rising worry regarding the toxicity of these substances to both animals and humans. For example, large amounts of TNP and Cd can be found in sewage sludge, surface water, and soil [16, 17]. Additionally, TNP have a history of significant discharge into water sources because of their usage in different industrial activities [18]. Cd is also the most pervasive pollutant in water systems [19]. Research on the effects of TNP-Cd co-exposure has mostly focused on plants [20] and non-mammalian animal models [21]. Still, little is known about the toxicological effects of TNP and Cd in mammals [22].

Oxidative damage and its complications are the central mechanisms of TNP [23] and Cd [24] nephrotoxicity; hence, supplementation with antioxidants may protect against their negative impacts. A growing number of studies have highlighted the need to utilize natural antioxidants to mitigate oxidative damage to the kidneys caused by exposure to environmental contaminants [25–27]. Coenzyme Q (CQ) is a naturally occurring molecule chiefly present in the phospholipid bilayer of the mitochondrial inner membranes, but it is also located in other biological membranes and plasma lipoproteins [28]. CQ9 is the predominant form in mice and rats, whereas, in humans and other long-living mammals, the major homolog is CoQ10 [29]. Yet, CQ10 supplementation in rats significantly increases CQ9 levels in the spleen and liver [30]. CQ10 is known for its antioxidant properties by enhancing mitochondrial function and warding off free radical damage [31]. However, certain redox-active metals like iron, copper, chromium, and vanadium can interact with CQ10 and potentially affect its antioxidant activity [32]. Yet, Cd is not redox-active and consequently does not affect CQ10 antioxidant potency. Besides, the meta-analysis performed by Bakhshayeshkaram et al. [33] concluded that CoQ10 supplementation in patients with chronic renal disease may improve aspects of their metabolic profiles, such as creatinine, lipid parameters, and oxidative damage markers. Also, CQ10 significantly protected rat kidneys against oxidative stress caused by various toxins and pharmaceuticals [34, 35]. Over other antioxidants, CQ10 has extra advantages in terms of 5–10 times more concentrated than other fat-soluble antioxidants like vitamin E and effectively inhibits pre-oxidant activities [36]. CoQ10 reduces α-tocopheroxyl radicals to α-tocopherol, which regenerates vitamin E and its functionality, indicating an indirect influence on enhancing antioxidant capacity [37]. Yet, the half-life of CQ10 in plasma is only about 33 h, and steady administration of CQ10 is needed for chronic oxidative diseases [38]. CQ10 has shown potential in decreasing Cd toxicity by enhancing antioxidant defenses in various animal models [39, 40]. Recently, CQ10 significantly mitigated the Cd-induced hepatorenal damage [41]. Hence, we hypothesized CQ10’s daily administration could mitigate impaired renal function caused by TNP and Cd co-exposure.

Therefore, the primary objective of this experiment was to compare the effects of rats exposed to both TNP and Cd individually with those exposed to the two substances alone on their kidney function. We further postulated that CQ10, due to its biological activities, could mitigate the harmful effects of TNP and Cd exposure on the kidney.

## Materials and Methods

### Tested Compounds

Alpha Chemica of Mumbai, India, supplied TNP) 99.98% purity, M.W = 79.87) and cadmium chloride (MW = 183.32). The sizes and morphology of the TNP were determined using a scanning electron microscope (SEM, JSM-6701F Plus, JEOL) and a transmission electron microscope (TEM-2100, Japan). An atomic force microscope (AFM, 5600LS, Agilent) was used to get the three-dimensional (3D) topography pictures of the TNP. We also used a zeta analyzer (Nano Sight NS500, Malvern Instruments Ltd.) and dynamic light scattering (DLS) to investigate the samples’ zeta potential (ZP) and particle size, respectively. An X-ray diffractometer like an XRD, D8-Discover, Bruker, or CuKα radiation was used to examine the crystal and phase structure. The TNP characteristics were detailed in our recent work [42], which demonstrated their homogeneous spherical shape, with particle sizes ranging from 6 to 19 nm. The study also confirmed the rutile crystal type through its characteristic XRD pattern, while the hydrodynamic diameter of the TNP was 12 nm, accompanied by a zeta potential of − 25 mV.

Mepaco-Medifood Co. of Cairo, Egypt, supplied the CQ10. The sterile distilled water was used to prepare the TNP solution freshly. Distilled water and corn oil (Arma Food Industries of Sharkia, Egypt, on the 10th of Ramadan) were used to dissolve CdCl_2_ and CQ10, respectively. Sigma-Aldrich Co. of St. Louis, MO, USA, supplied the remaining analytically grade chemicals and reagents.

### Experimental Animals

A group of 70 adult male Sprague Dawley rats, 155.58 ± 0.52 g and 10 weeks old, were procured from the breeding section of the National Research Centre in Giza, Egypt. Stainless steel cages were used to house the animals, while hardwood shavings were used as bedding. Rat had free access to commercial standard rat chow (El Gomhouria Company, Cairo, Egypt) containing 65% carbohydrates, 20.3% proteins, 5% fat, 5% fiber, 3.7% salt mixture, and 1% vitamin mixture. The animals were kept in a 12-h light–dark cycle and given water on demand. A 2-week acclimatization period was given to the rats before the start of the experiment.

Per the following regulations: the National Research Council’s Guide for the Care and Use of Laboratory Animals, the EU Directive 2010/63/EU for animal experiments, and the UK Animals (Scientific Procedures) Act, 1986 and associated guidelines, all animal experiments were permitted by the Ethics Committee of the Faculty of Veterinary Medicine, Cairo University, Egypt (Approval no. VET CU 2009 2022462). Besides, all methods were reported in line with ARRIVE guidelines [43].

### Experimental Outline

The rats were randomly allocated to seven sets, each including ten rats. Each rat in the control, vehicle control (VC), and CQ10-treated groups was orally given 1 mL distilled water, 1 mL corn oil, or 10 mg of CQ10 (dissolved in corn oil)/kg b.wt, respectively. The TNP-exposed group was given an oral suspension at 50 mg TNP /kg b.wt. The Cd-exposed group received an oral dose of 5 mg Cd/kg b.wt. The TNP and Cd co-exposed group received both TNP and Cd orally at the aforementioned levels. Moreover, the TNP, Cd, and CQ10 co-treated group received TNP, Cd, and CQ10 orally at the doses above. The dosing lasted for 60 consecutive days using oral gastric gavage.

### TNP, Cd, and CQ10 Dose Selection

The current study’s tested dose was determined using the European Food Safety Authority (EFSA) suggested dosages for oral consumption of food-grade TiO_2_ (E171) and TNP. Although the EFSA panel has yet to nominate an acceptable daily intake for E171 [44], EFSA [45] reports that in the maximum E171exposure scenario, the average daily exposure for adults is 0.6–6.8 mg/kg b.wt, but for children, it ranges from 1.8 to 10.4 mg/kg b.wt. Based on Nair and Jacob [46], when applied to the rat dose, the dosage ranges from 41.98 to 64.20 mg/kg b.wt daily. Similarly, E171 includes around 36% TNP with a less than 100-nm diameter [47]. At the 95th percentile, adults’ maximum TNP exposure levels were assessed to be 0.07 to 0.48 mg/kg b.wt/day [45]. In the current study, to calculate the exposure dose and account for the possibility of extrapolating between species, as well as the many sources of exposure, we used a safety factor of 100. [48]. As a result, a TNP dose of 50 mg/kg bw was selected in rats, which is 100 times the dose of the anticipated maximum exposure in adults.

The chosen dose for Cd was shown to cause significant oxidative stress in numerous organs of the body [49–51]. The dose of CQ10 was chosen based on prior research that revealed the anti-oxidant, anti-apoptotic, and anti-inflammatory characteristics of CoQ10 at this dose in several rat organs [52–54].

### Sampling

The rats in each group were given 100 mg pentobarbital sodium/kg b.wt. intraperitoneally after a 24-h fast, weighed, and anesthetized. The blood from the retro-orbital venous plexus was taken into a simple tube and left at room temperature for 20 min. The tubes were then centrifuged for 10 min at 3000 rpm, and the serum was stored at − 20 °C for future biochemical analysis. The rats were subsequently decapitated, and the kidneys were necropsied, rinsed with saline, and weighed. The kidney samples were split into three sets. The first group was fixed in a 10% buffered neutral formalin solution for histopathological analysis. The second set of kidney samples underwent homogenization using a Potter–Elvehjem rotor–stator homogenizer Thomas Scientific, Swedesboro, NJ, USA), in an ice-cold phosphate buffer saline. The resulting homogenate was centrifuged for 10 min at 4 °C and 3000 rpm. The supernatants were then evaluated for the biochemical tests outlined below. The last one was kept at 4 °C until the metal (Cd and Ti) content was determined.

### Assessment of Serum Kidney Function Parameters and Electrolytes

Spinreact kits (Sant Esteve De Bas, Spain) with catalog no. MDBSIS45-I, BSIS35-I, and BSIS13-I were used to measure uric acid, urea, and creatinine serum levels, respectively. Total serum proteins and albumin were measured by Spinreact kits with catalog no. BSIS30-I and BSIS02-I, respectively. The globulin concentration was determined by subtracting the albumin content from the total protein content. Serum sodium (Na), potassium (K), and calcium (Ca) content were determined using Spinreact kits with catalog no. BSIS54-I, BSIS53-I, and MDBSIS09-I, respectively.

### Evaluation of Lipid Peroxidation and Renal Oxidative Stress

Using biodiagnostic colorimetric bioassay kits (Dokki, Giza, Egypt) with catalog no. SD 25 21 and MD 25 29, the superoxide dismutase (SOD) and malondialdehyde (MDA) contents were evaluated in the kidney homogenates. Bio-Assay Systems’ EnzyChromTM (CA, USA), glutathione Peroxidase Assay Kit (EGPX-100) was used to evaluate glutathione peroxidase (GPx) activity.

### Analysis of the Renal Content of Cd and Ti

We used 8 mL of nitric acid, 1 mL of hydrogen peroxide (H_2_O_2_), and 30% of the sample volume to digest the kidney samples in the microwave. An inductively coupled plasma-optical emission spectrometer was used to determine the amounts of Ti and Cd. This instrument, a model 5100 from Agilent in Santa Clara, CA, was equipped with a Synchronous Vertical Dual View. An intensity calibration curve was generated for every set of measurements by a blank and a minimum of three standards from the Merck Company. To ensure the quality and precision of the metal analysis, external Merck reference standards were used. In addition, trace element quality control samples were relative to NIST standards to validate the instrument’s reading. By matching certified values with analyses of the reference sample, recovery rates for Cd were found to be 98% and for Ti to be 97%.

### Histopathological Evaluations

From each animal, the right kidney tissue samples were prepared following Bancroft and Layton [55] technique. After formalin fixation and dehydration with increasing alcohol concentrations, they were rinsed with xylene, embedded in paraffin, and then sealed. Then, they were sectioned of 5-μm thickness and stained with hematoxylin and eosin (H &E). The sections were also stained with Masson’s trichrome stain to demonstrate collagen fibers [56].

### Statistical Analysis

The Kolmogorov–Smirnov and Levene’s tests were employed to certify that the data was normally scattered with homogeneous variances. When normality criteria were met, the data was examined with a one-way analysis of variance (ANOVA) in SPSS version 14. (SPSS, Chicago, IL, USA). Following this, Tukey’s multiple range post hoc test was applied for group comparisons in cases where normality assumptions were satisfied. For the metal residue findings, a Kruskal–Wallis test followed by Dunn’s multiple comparisons test was applied. A *p*-value of less than 0.05 was used to outline significance.

## Results

### Changes in Renal Function Indices

**Fig. 1:**
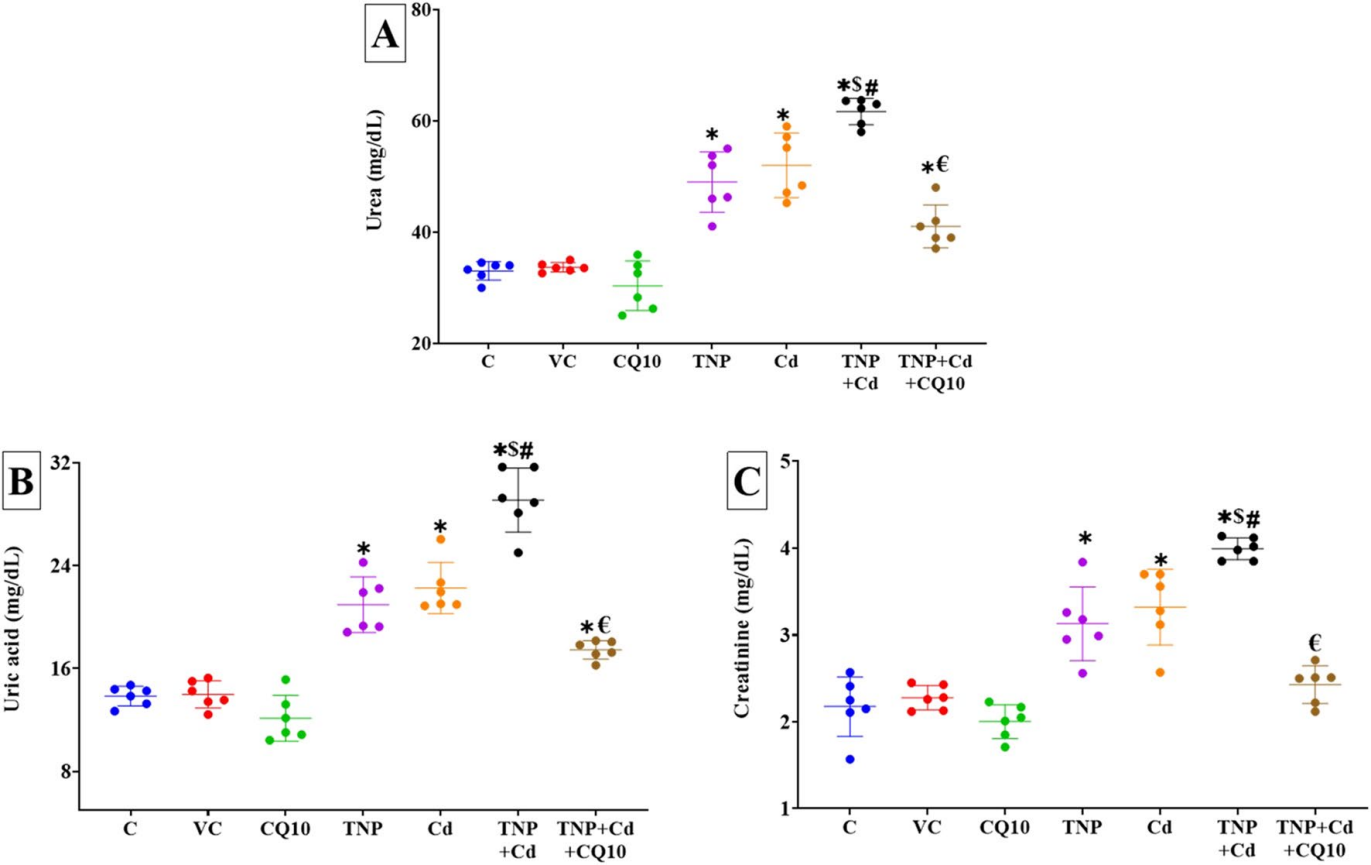
Effects of coenzyme 10 (CQ10) on serum levels of **A** urea, **B** uric acid, and **C** creatinine of rats exposed to titanium dioxide nanoparticles (TNP) and/or Cd for 60 days. Data are expressed as the mean ± SD (*n* = 6). *p < 0.05 vs control, $*p* < 0.05 TNP + Cd vs Cd, #*p* < 0.05 TNP + Cd vs TNP, and €*p* < 0.05 TNP + Cd + CQ10 vs TNP + Cd

The serum levels of urea, uric acid, and creatinine were significantly (*p* < 0.001) increased after the 60-day single or mutual exposure to Cd and TNP than the control group (Fig. 1A–C). Remarkably, the TNP and Cd co-exposed group exhibited a significant increase in the analyzed renal damage products compared to the single TNP or Cd-exposed group. Instead, the serum levels of urea, uric acid, and creatinine were significantly lower in the TNP + Cd + CQ10 co-treated group than in the TNP and Cd co-exposed group. The creatinine levels in the serum did not change significantly between the control group and the group that received a combination of TNP, Cd, and CQ10 (Fig. 1C).

### Changes in the Serum Protein Profile

**Fig. 2:**
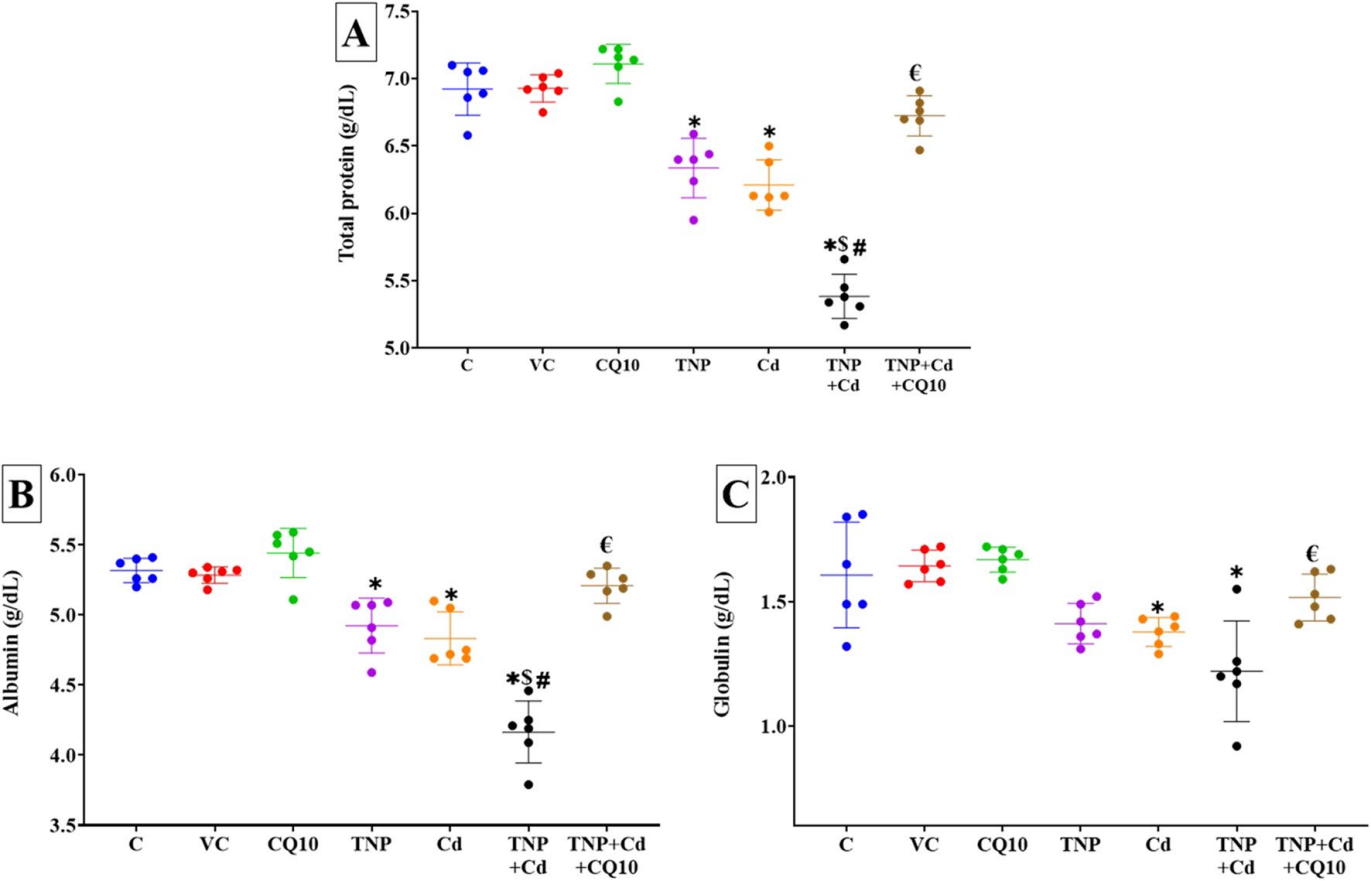
Effects of coenzyme 10 (CQ10) on serum levels of **A** total protein, **B** albumin, and **C** globulin of rats exposed to titanium dioxide nanoparticles (TNP) and/or Cd for 60 days. Data are expressed as the mean ± SD (*n* = 6). *p < 0.05 vs control, $*p* < 0.05 TNP + Cd vs Cd, #*p* < 0.05 TNP + Cd vs TNP, and €*p* < 0.05 TNP + Cd + CQ10 vs TNP + Cd

After 60 days of oral administration of TNP and/or Cd, as well as CQ10, the protein profile changed in rats (Fig. 2A–C). Compared to the control group, the groups exposed to TNP and/or Cd had significantly lower serum levels of total protein and albumin (*p* < 0.001). Also, the Cd and Cd + TNP-exposed groups had significantly lower serum levels of globulin (*p* < 0.001). Notably, serum albumin levels were significantly lower in rats exposed to both TNP and Cd simultaneously compared to rats treated to either compound alone. Total protein, albumin, and globulin serum levels were substantially restored in the TNP + Cd + CQ10 co-treated group compared to the TNP and Cd co-exposed group. Substantial (*p* < 0.001) improvements in the protein profile were attained with oral administration of CQ10, to the point that the control groups did not show any changes.

### Effects on Electrolyte Balance

**Fig. 3:**
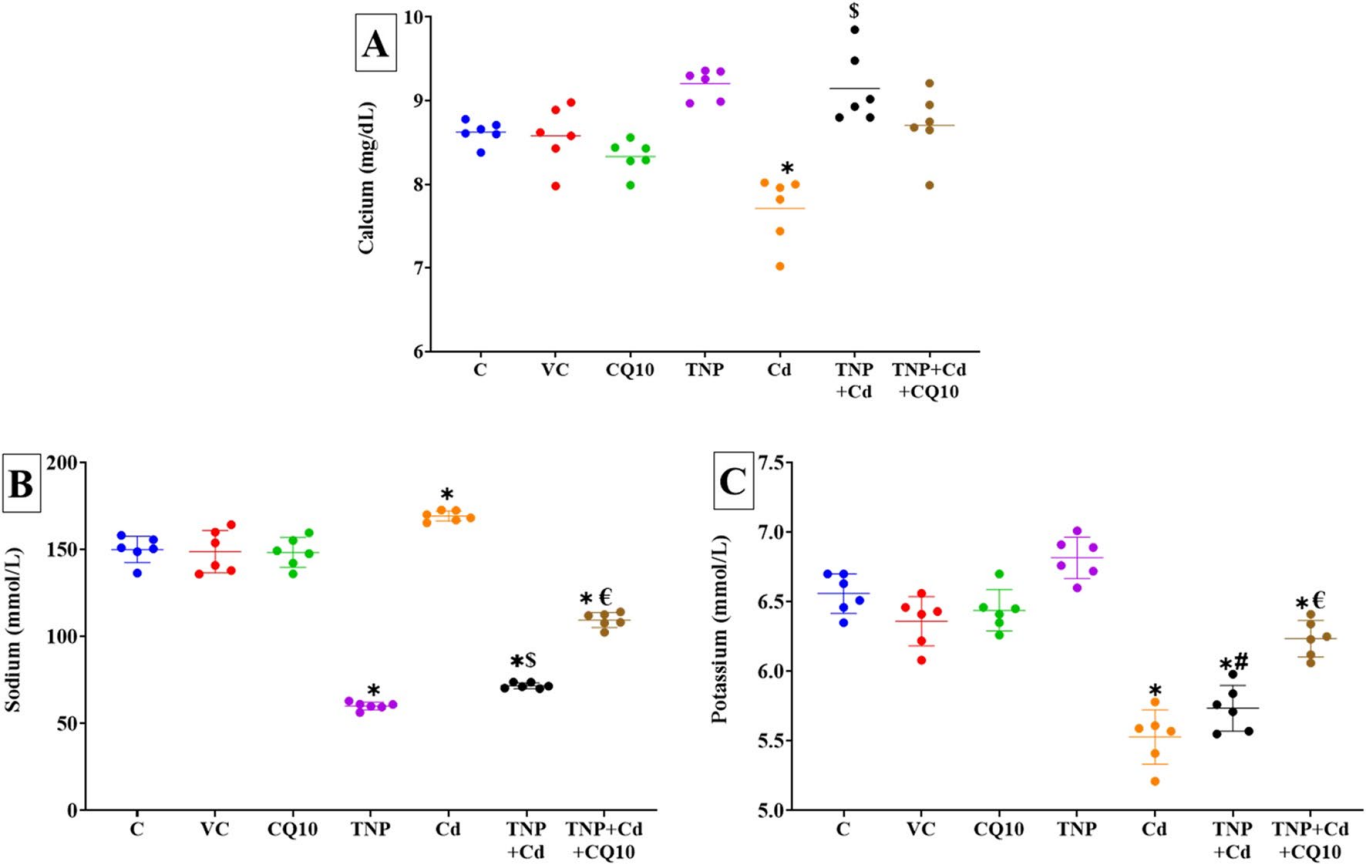
Effects of coenzyme 10 (CQ10) on serum levels of **A** calcium, **B** sodium, and **C** potassium of rats exposed to titanium dioxide nanoparticles (TNP) and/or Cd for 60 days. Data are expressed as the mean ± SD (*n* = 6). *p < 0.05 vs control, $*p* < 0.05 TNP + Cd vs Cd, #*p* < 0.05 TNP + Cd vs TNP, and €*p* < 0.05 TNP + Cd + CQ10 vs TNP + Cd

Regarding changes in serum Ca^2+^ level, as demonstrated in Fig. 3A, the groups exposed to TNP or TNP and Cd displayed a significant (*p* < 0.001) increase and a tendency to increase, respectively in their level compared to the control group. Nonetheless, the Cd-rats displayed a significantly (*p* < 0.001) lower serum Ca^2+^ level than the control ones. Nonetheless, the TNP + Cd + CQ10 co-treated group exhibited a tendency to reduction in serum Ca^2+^ levels than the TNP and Cd co-exposed group. Additionally, no significant changes were recognized in the serum Ca^2+^ level between the control and TNP + Cd + CQ10 co-treated groups.

Concerning changes in serum Na^+^ level, as demonstrated in Fig. 3B, the Cd-exposed rats displayed a significant rise than the control ones. Nonetheless, a significant reduction in serum Na^+^ level was found TNP or TNP and Cd-exposed group compared to the control group. However, the TNP + Cd + CQ10 co-treated rats displayed a significant increase in serum Na^+^ levels than the TNP and Cd co-exposed group. As displayed in Fig. 3C, the groups exposed to Cd or TNP and Cd showed a significant drop in serum K^+^ level than the control group. A tendency toward increase was recorded in the TNP-exposed rats compared to the control group. However, the TNP + Cd + CQ10 co-treated rats exhibited a significant increase in serum K^+^ level relative to the TNP and Cd co-exposed group.

### Changes in Renal Oxidative Status

**Fig. 4:**
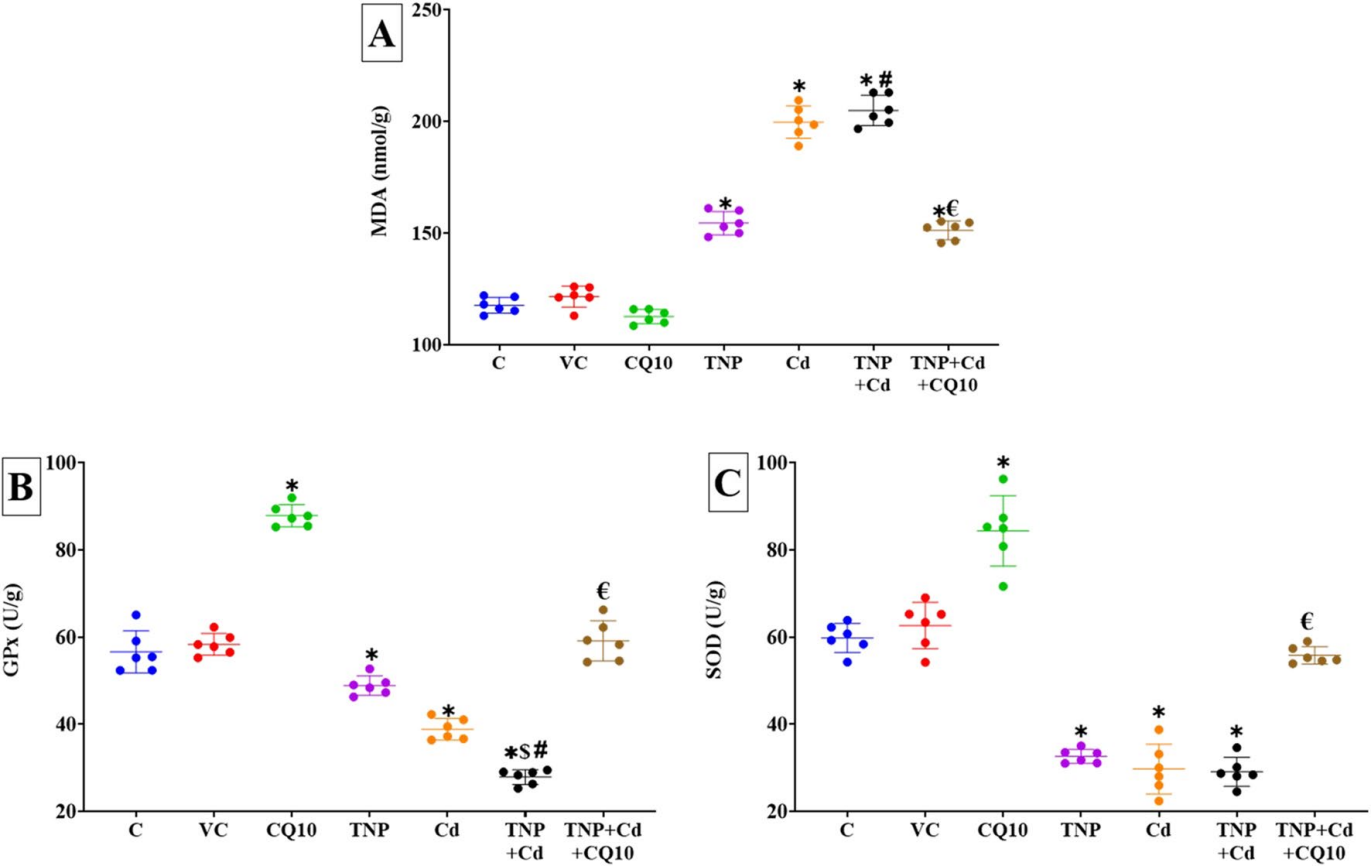
Effects of coenzyme 10 (CQ10) on antioxidant enzymes and lipid peroxidation indicators including **A** malondialdehyde (MDA), **B** glutathione peroxidase (GPx), and **C** superoxide dismutase (SOD) indicators in kidney homogenate of rats exposed to titanium dioxide nanoparticles (TNP) and/or Cd for 60 days. Data are expressed as the mean ± SD (*n* = 6). *p < 0.05 vs control, $*p* < 0.05 TNP + Cd vs Cd, #*p* < 0.05 TNP + Cd vs TNP, and €*p* < 0.05 TNP + Cd + CQ10 vs TNP + Cd

The CQ10 oral dosing significantly (*p* < 0.001) raised SOD and GPx activities but decreased MDA content in the kidney tissues when than the control group, as demonstrated in Fig. 4A–C. In contrast, rats exposed to TNP and/or Cd for 60 days showed a notable decrease in SOD and GPx antioxidant enzymes but a rise in MDA compared to the control group, with statistically significant exhaustion of these enzymes (*p* < 0.001). Importantly, GPx levels differed significantly between rats treated with Cd alone or co-exposed to TNP and Cd. While both SOD and GPx levels were noticeably greater in the TNP + Cd + CQ10 co-treated group compared to the TNP and Cd co-exposed group, MDA levels were noticeably lower in the latter. Surprisingly, no significant change in SOD and GPx activities was recorded between CQ10 + TNP + Cd co-exposed group and control group.

### Effects on Ti and Cd Renal Accumulation

**Fig. 5:**
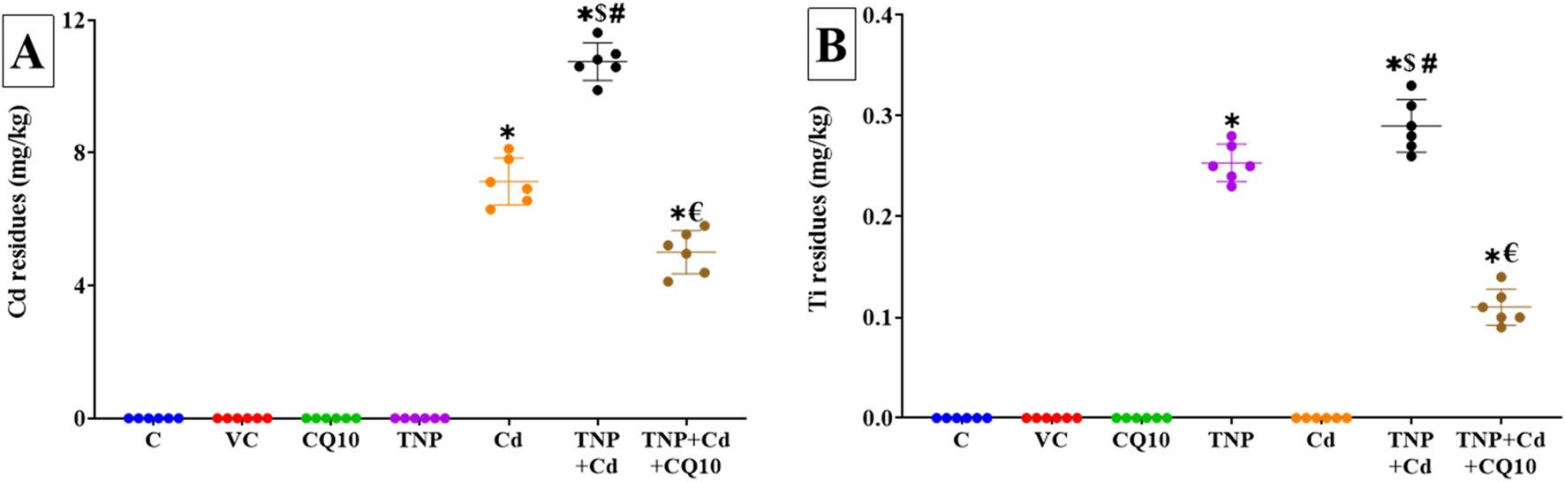
Effects of coenzyme 10 (CQ10) on cadmium (Cd) and titanium (Ti) residues in kidney homogenate of rats exposed to titanium dioxide nanoparticles (TNP) and/or Cd for 60 days. Data are expressed as the mean ± SD (*n* = 6). *p < 0.05 vs control, $*p* < 0.05 TNP + Cd vs Cd, #*p* < 0.05 TNP + Cd vs TNP, and €*p* < 0.05 TNP + Cd + CQ10 vs TNP + Cd

The control, VC, CQ, and TNP groups had undetectable concentrations of Cd in their renal tissues (Fig. 5A). Yet, rats orally given Cd and those co-administered Cd and TNP had significant (*p* < 0.001) concentrations of Cd than the rats in other experimental groups. Nonetheless, the Cd + TNP co-exposed group had significantly (*p* < 0.001) higher renal Cd levels than the Cd-exposed group. Yet, the renal Cd content was significantly (*p* < 0.001) lower in the TNP + Cd + CQ10 co-treated group than in the TNP and Cd co-exposed group. Concerning renal Ti accumulation, compared to the control group, the groups exposed to TNP or TNP + Cd showed significantly (*p* < 0.001) higher Ti content (Fig. 5B). Rats exposed to both TNP and Cd showed greater renal Ti concentration compared to rats exposed to TNP alone. Contrary to the TNP + Cd co-exposed group, the oral dosage of CQ10 dramatically reduced renal Ti accumulation (*p* < 0.001).

### Histopathological Findings

**Fig. 6:**
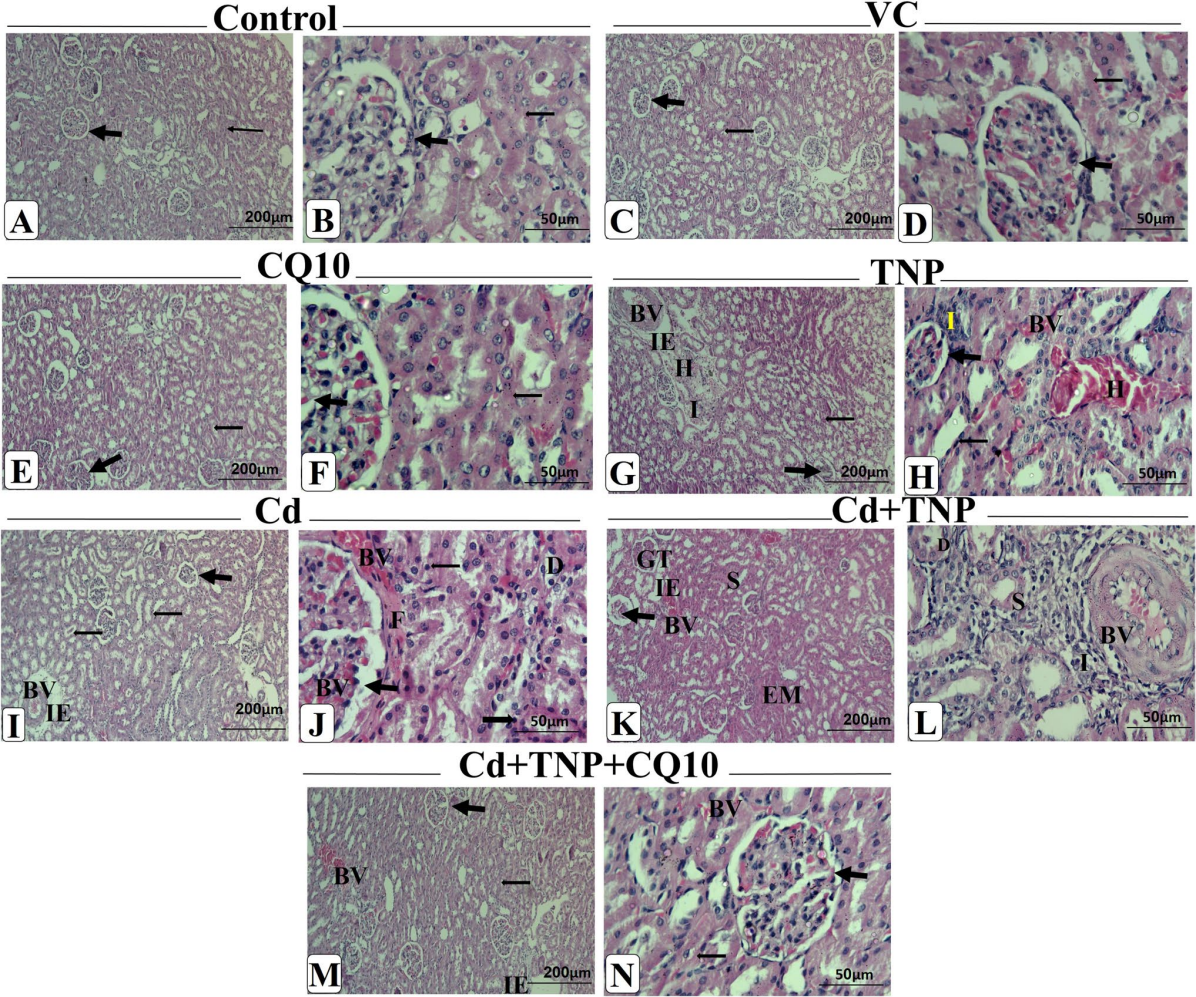
Photomicrograph of the H&E-stained kidney sections of different experimental groups. Control (**A**, **B**), vehicle control (VC) (**C**, **D**), and co-enzyme Q10 (CQ10) (**E**, **F**) groups showing normal renal tubules and glomeruli (H&E, × 100 and 400 × , respectively). Titanium dioxide nanoparticle-exposed (TNP) group showing (**G**) chronic interstitial glomerulonephritis. Some tubules showed dilatation with the presence of desquamated or degenerated epithelial cells, glomeruli shrinkage associated with dilated Bowman’s space, interstitial edema, hemorrhage, congestion of interstitial blood vessels with, as well as focal leucocytic infiltrations and interstitial fibrosis (H&E, × 100), (**H**) dilated tubules with desquamated or degenerated epithelial cells, shrinked glomeruli with dilated Bowman’s space, hemorrhage, congested interstitial blood vessels with as well as focal leucocytic infiltrations (H&E, × 400). Cadmium chloride (Cd)-exposed group showing (**I**) some tubules dilated and others had degenerated epithelial cells, glomeruli shrinkage associated with dilated Bowman’s space, interstitial edema and congested intertubular blood vessels with vasculitis (H&E, × 100), (**J**) degenerated epithelial cells of tubules, desquamated epithelium of tubules, glomeruli shrinkage and congested associated with dilated Bowman’s space and fibrosis around glomeruli as well as congested intertubular blood vessels (H&E, × 400). Cd + TNP co-exposed group showing (**K**) cloudy swelling of renal tubules, tubular eosinophilic material, congestion of glomerular tuft, congested blood vessels and interstitial edema (H&E, × 100), (**L**) interstitial nephritis with inflammatory cell infiltrations, desquamated epithelium, cellular swelling with pyknotic nuclei, vacuolated real epithelium and congestion of interstitial blood vessels with thickened wall and vasculitis (H&E, × 400). Cd + TNP + CO10-treated group showing (**M** and **N**) normal structure with normal renal tubules and glomeruli and mild congestion of blood vessels and interstitial edema (H&E, × 100 and 400 × , respectively). Abbreviations: Thin arrow, renal tubule; thick arrow, glomeruli; BV, congestion of blood vessels; IE, interstitial edema; H, hemorrhage; I, leucocytic infiltrations; S, cloudy swelling; EM, tubular eosinophilic material; GT, glomerular tuft; D, desquamated epithelium; F, fibrosis

The microscopic investigation of the normal control group revealed the normal histological structure of the kidney tissues’ outer cortex and inner medulla. There were well-developed glomeruli proximal and distal convoluted tubules without any alterations (Fig. 6A and B). Also, there was no histological difference between VC (Fig. 6C and D) and CQ10-treated groups, which were similar to the control group (Fig. 6E and F). On the other side, the kidneys of groups treated with TNP and/or Cd showed remarkable chronic interstitial nephritis with degenerative changes (Fig. 6G–J). Some tubules showed dilation with desquamated or cytoplasmic vacuolations of tubular epithelial cells, while others had cloudy swelling and narrowing of the lumen. Also, some tubular cells had pyknotic nuclei. Glomeruli shrinkage associated with dilated Bowman’s space, thickened basement membranes, and congestion of glomerular tufts were observed. There was prominent focal interstitial hemorrhage and edema. Congestion and dilatation in the interstitial blood vessels associated with vasculitis were noted. Interstitial inflammatory cell infiltrations with variable numbers of lymphocytes, macrophages, and plasma cells were seen. There was focal interstitial and perivascular fibrosis. The severity of the previously histopathological alterations was prominently observed in a co-exposure group (Fig. 6K and L). The co-exposure group that was treated with CQ10 showed restoring the normal histological structure. Renal tubules and glomeruli were more or less normal, but a few tubules revealed cytoplasmic vacuolations and mild edema in their epithelial lining cells (Fig. 6M–N).

### Histochemical Results

**Fig. 7:**
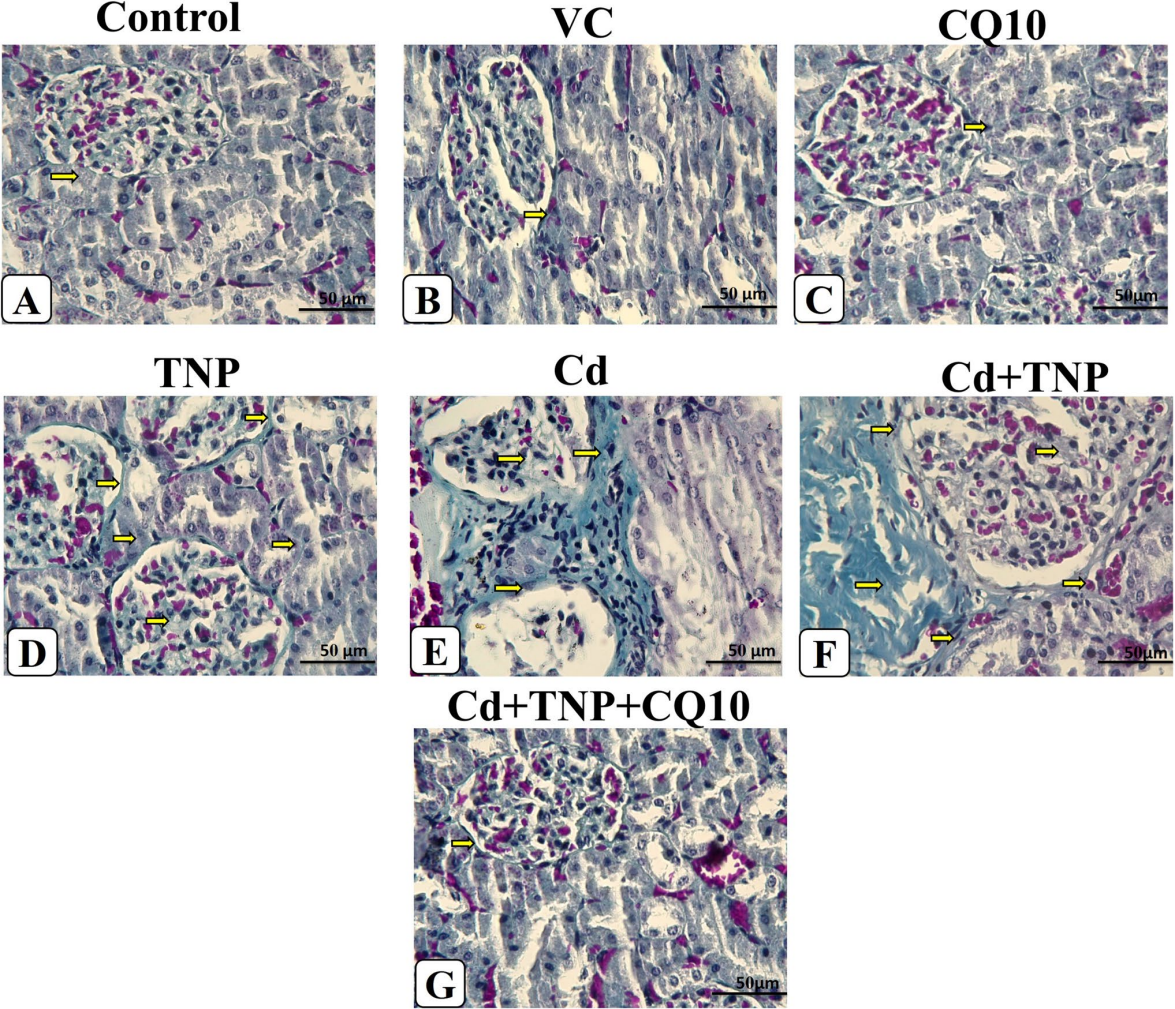
Photomicrograph of kidney tissue sections of different experimental groups stained with Masson’s trichrome identified by the bluish color of collagen fibers in the renal corpuscle and between the renal tubules (arrows). The control group (**A**), vehicle control-(VC, **B**), and co-enzyme 10-treated (CQ10, **C**) groups showed normal density and distribution of collagen fibers. Titanium dioxide nanoparticle-exposed (TNP) group showing (**D**) wide areas of positive results as periglomerular, peritubular, perivascular, and interstitial tissue collagen deposition. Cadmium chloride-exposed (Cd, **E**) group showing an increase in the deposition of collagen fibers. TNP + Cd co-exposed group showing (**F**) increased fibrous tissue inside glomeruli, interstitial tissue, periglomerular, peritubular, and perivascular. Cd + TNP + CO10 co-treated group showing (**G**) a normal pattern of collagen deposition (Masson’s trichrome stain, × 400)

Sections from control, CQ10, and VC-treated groups revealed that Masson’s trichrome stain distinguished the normal distribution of collagen fibers. Collagen staining appeared in a small amount around tubules and the vessels and was rare in the glomeruli (Fig. 7A, B, and C). TNP and/or Cd showed marked deposition of collagen fibers in periglomerular, peritubular, perivascular, and interstitial tissue and also occurred in some glomeruli (Fig. 7D, E, and F). In the co-exposure group with CQ10 treatment, kidneys revealed lower collagen staining than the TNP and Cd co-exposed group (Fig. 7G).

## Discussion

Several earlier studies have identified impaired serum urea, uric acid, and creatinine as reliable nephrotoxicity indicators [25, 57, 58]. Here, after 60 days of Cd and/or TNP oral dosing, urea, uric acid, and creatinine levels considerably increased, signifying severe renal damage. These results were confirmed by the histological and histochemical observations, which showed that prominent chronic interstitial nephritis and degenerative changes together with marked deposition of collagen fibers periglomerular, peritubular, perivascular, and interstitial tissue of groups treated with TNP and/or Cd. Collagen is the major insoluble fibrous protein in the extracellular matrix that could be distinguished by Masson’s trichrome stain and its increasing reflected cellular damage [59]. Injuries to the kidneys caused by accumulated Cd or TNP trigger the activation of inflammatory cells within the glomeruli or interstitial spaces. These cells then release cytokines that are inflammatory, fibrogenic, and ROS [60, 61]. These trigger a cascade of events that increase the formation of extracellular matrix components through routes such as stimulation of mesangial and fibroblast cells and tubular epithelial-to-mesenchymal transition [61, 62]. Kidney fibrosis can develop from this accumulation and deposition, which is too much for the kidneys to handle. This could be mainly related to the reduced accumulation of Ti and Cd in the CQ10-treated groups, probably due to its chelating activity [63]. Additionally, the antioxidant activity of CQ10 could be responsible for maintaining the tubular architecture and preventing the leak of urea, uric acid, and creatinine into the blood [64]. Moreover, Jiang et al. [65] reported that CQ10 may reduce renal fibrosis through multiple mechanisms; however, its antioxidant capability may be its main contributing element.

Noticeable hypoproteinemia, hypoalbuminemia, and hypoglobulinemia were recorded in Cd and/or TNP-treated rats. Several factors can contribute to the decreased serum total protein, globulin, and albumin, levels in Cd and/or TNP-induced kidney damage like proteinuria and concomitant loss of albumin and other proteins from the bloodstream, leading to decreased serum total protein, albumin, and globulin levels [66]. Also, a strong binding of TNP with different cellular proteins was observed in the in silico docking study of Ranjan et al., [67] could reduce the ability of the kidney to produce proteins at the normal rate. Importantly, albumin and most of the proteins in the globulin fraction, which comprises carrier proteins, complement, immunoglobulins, and enzymes are synthesized in the liver [68]. Hence, the previously documented TNP and Cd-induced hepatic injury could be highly responsible for the depleted serum proteins [42]. The Cd and TNP-induced alteration of protein metabolism could also be another probable cause [69, 70]. Interestingly, herein, Cd and TNP-induced kidney damage was associated with inflammatory reactions detected histologically by the interstitial inflammatory cell infiltrations in the renal tissue. This can affect the production and regulation of various proteins, including albumin and globulins, leading to decreased levels [71, 72]. In contrast, CQ10 oral dosing considerably corrected the Cd and TNP-induced reduction in serum protein levels and increase in interstitial inflammatory cell infiltrations. These findings suggest that CQ10 can mitigate inflammatory alterations in the kidneys. Additionally, the serum protein restoration with CQ10 dosing could be highly linked to its hepatoprotective activity [68].

Healthy mineral balance is essential for proper kidney function [73]. Disorders in the concentrations of serum electrolytes, including Na^+^, K^+^, and Ca^2+^, are endorsed to disturb kidney function [74]. In the current study, each Cd and TNP induced distinct alterations in the electrolyte balance. TNP-treated rats showed significant hypercalcemia, hyponatremia, and hyperkalemia, while Cd-treated rats displayed significant hypocalcemia, hypernatremia, and hypokalemia. The in situ quantitative study of Simon et al. [75] stresses that TNP highly altered the intracellular Ca^2+^ homeostasis. Also, Bian et al. [76] recently confirmed the role of increased intracellular Ca^2+^ in the pathological effects of TNP and attributed this increase to the perturbation of membrane phospholipids. The disruption of the cytoplasmic membrane by TNP could alter the function of sodium-glucose cotransporters and ion cotransporters, mainly the Na^+^-K^+^-Cl cotransporter. These transporters are the major participants in urine osmolarity. The effects would be due to the dysregulation of these nephron cotransporters [77]. Cd increased the serum Na^+^ and decreased the K^+^ levels. This could be because of an alteration in Na^+^/K^+^-ATPase activity and the intracellular and intercellular Na^+^ and K^+^ distribution in response to Cd [78]. Serum Ca^2+^ levels decreased, probably due to the blockage of Ca^2+^ transport by Cd [79]. Notably, the co-exposure of TNP and Cd seemed to produce a unique pattern of electrolyte imbalance characterized by hypercalcemia, hyponatremia, and hypokalemia. This distinct electrolyte profile suggests a potential interaction between TNP and Cd in disrupting ion transport mechanisms. The specific mechanisms underlying these electrolyte imbalances may involve complex interactions and pathways that require further investigation. Additionally, the observed differences in the electrolyte profiles between the TNP + Cd co-exposed group and the individual TNP or Cd-exposed groups indicate potential synergistic or additive effects of these substances on electrolyte homeostasis. Instead, TNP + Cd + CQ10-treated rats displayed a significant correction of the altered electrolyte profile. CQ10 has been suggested to be beneficial in correcting electrolyte imbalances due to its essential role in cellular energy production, mitochondrial function, and antioxidant properties. CQ10 is an essential component of the mitochondrial electron transport chain, where it helps generate adenosine triphosphate (ATP), the primary energy currency of cells [80]. Proper ATP production is crucial for maintaining the normal functioning of ion channels, transporters, and pumps involved in electrolyte balance regulation [81]. By supporting mitochondrial function, CQ10 may indirectly promote the normal activity of these important cellular processes. CoQ10 possesses antioxidant properties, meaning it has the potential to lessen oxidative stress and protect against damaging free radicals [82]. Electrolyte imbalances can be influenced by oxidative stress-induced damage to cells, including those responsible for electrolyte transport [83]. CQ10 may help protect these cells from damage by reducing oxidative stress, improving electrolyte regulation. CQ10 has been found to modulate cellular signaling pathways and gene expression, which can influence the activity of ion channels, transporters, and pumps involved in electrolyte balance [84]. By regulating gene expression and signaling cascades, CoQ10 may help restore the normal function of these components, promoting the correction of electrolyte imbalances.

TNP and Cd’s ability to generate excessive ROS and free radicals was assumed to be the main cause of tissue injury [85, 86]. This was evident in the present study’s results, which demonstrated that rats exposed to TNP and/or Cd had considerable exhaustion of antioxidant enzymes yet a noticeable MDA accumulation. In contrast, single CQ10 treatment significantly elevated SOD and GPx activity in renal tissues than the control. Furthermore, CQ10 dramatically reduced TNP and Cd-induced oxidative stress. CQ10 is a naturally occurring lipid-soluble antioxidant that keeps DNA, lipids, and proteins from oxidation [87]. CQ10 could indirectly decrease oxidative stress through improving the efficacy of mitochondria’s electron transport chain, defending against the damage of uncontrolled electrons, and promoting the recovering of other antioxidants [88]. Furthermore, CQ10’s ability to neutralize singlet oxygen and peroxy radicals makes it an effective tool in the battle against ROS [89]. Many human investigations have found that CQ10 has a much stronger antioxidant capacity than vitamin C or E [90, 91].

In the present investigation, rats separately exposed to TNP or Cd accumulated significantly more Ti and Cd in their renal tissues. Numerous studies on Cd toxicity have established that the kidney is a primary organ site for Cd accumulation [92, 93]. Besides, Orr and Bridges [94] reported that up to 50% of the body’s Cd pool can be deposited in the kidney because of its metallothionein content. Previous toxicokinetic investigations demonstrated that repeated oral TNP treatment could harm and pass across the intestinal barrier, accumulate in numerous tissues, particularly the kidney, and have deleterious consequences [95]. Furthermore, rats exposed to TNP and Cd exhibited greater renal Ti and Cd renal levels than rats exposed to either pollutant alone. NP-assisted heavy metal ion buildup in mammals’ organs could increase toxicity in vivo under co-exposure settings [8, 96, 97]. In this respect, Li [22] postulated that TNP may operate as a transporter for Cd entering cells, increasing its accumulation. Furthermore, TNP-Cd nanoadducts may have a lower clearance rate than free Cd ions. TNP-induced cell membrane disruption and increased vascular permeability may accelerate Cd uptake. On the contrary, oral treatment of CQ10 dramatically lessened the buildup of Cd and Ti in rat renal tissues. CQ10 has been shown to reduce heavy metal burdens in numerous tissues, implying that it may have excretion-enhancing or chelating properties [63]. In this regard, Ki et al. [98] reported that supplemental CQ10 increases its level in the plasma, but not in tissues. Yet, in the study of Ibrahim et al. [30] in rats, CQ10 exogenous administration markedly increased its concentration in the liver. Yet, the accumulation of CQ10 has been little investigated. Hence, future research could include monitoring plasma or renal CQ10 after exposure to a pollutant mixture to acquire a more comprehensive knowledge of CQ10’s potential protective effects.

Notably, co-exposure to both Cd and TNP renal products leakage, exhausted antioxidant enzymes, and raised MDA concentration more than individual exposure. These data revealed that mutual exposure to TNP and Cd resulted in greater functional and microstructural damage in the kidney. CQ10’s great antioxidant efficacy in keeping cell integrity from lipid peroxidation and ROS caused by hazardous chemicals could explain this result [99]. Importantly, despite the several estimated parameters assessing the renal function in this study, we acknowledge the need for further mechanistic studies providing a deep understanding of the other potentially involved pathways in the nephro-protective effects of CQ10, particularly the inflammatory cascade. Yet, despite this limitation, our study provides valuable evidence regarding the protective effects of CQ10 against Cd and TNP-induced nephrotoxicity, highlighting the potential for further research in this area.

## Conclusions

The negative effects of TNP and Cd exposure on renal function were demonstrated by the current study’s biochemical, histological, metal residues, and histopathological endpoints, especially when these two metals were exposed together. Furthermore, the results showed that CQ10 effectively protected the kidneys of rats exposed to both TNP and Cd simultaneously, likely due to its antioxidant properties. The alternative potential mechanisms of CQ10 and its destiny require additional research. The potential use of CQ10 as a protective approach in individuals prone to exposure to NPs/heavy metal mixtures, such as those working in the industrial sector, necessitates additional human investigations.

## Data Availability

Data is provided within the manuscript or supplementary information files.
